# The Role of Inflammation in the Pathogenesis of Comorbidity of Chronic Obstructive Pulmonary Disease and Pulmonary Tuberculosis

**DOI:** 10.3390/ijms26062378

**Published:** 2025-03-07

**Authors:** Stanislav Kotlyarov, Dmitry Oskin

**Affiliations:** 1 Department of Nursing, Ryazan State Medical University, 390026 Ryazan, Russia; 2 Department of Infectious Diseases and Phthisiology, Ryazan State Medical University, 390026 Ryazan, Russia

**Keywords:** COPD, pulmonary tuberculosis, inflammation, pathogenesis, innate immune system, adaptive immune system

## Abstract

The comorbid course of chronic obstructive pulmonary disease (COPD) and pulmonary tuberculosis is an important medical and social problem. Both diseases, although having different etiologies, have many overlapping relationships that mutually influence their course and prognosis. The aim of the current review is to discuss the role of different immune mechanisms underlying inflammation in COPD and pulmonary tuberculosis. These mechanisms are known to involve both the innate and adaptive immune system, including various cellular and intercellular interactions. There is growing evidence that immune mechanisms involved in the pathogenesis of both COPD and tuberculosis may jointly contribute to the tuberculosis-associated obstructive pulmonary disease (TOPD) phenotype. Several studies have reported prior tuberculosis as a risk factor for COPD. Therefore, the study of the mechanisms that link COPD and tuberculosis is of considerable clinical interest.

## 1. Introduction

The study of the comorbidity of chronic obstructive pulmonary disease (COPD) and pulmonary tuberculosis is currently of particular relevance due to the increasing burden of both diseases worldwide [1,2,3]. Widespread prevalence, frequent disability, and unfavorable prognosis in these diseases cause their significant contribution to the structure of morbidity and mortality of the population [4,5,6,7]. According to epidemiological studies, COPD is among the most significant causes of death, ranking third in this list. COPD is characterized by progressive intensification of airway obstruction and increasing respiratory failure due to chronic inflammation in the airways. This leads to the development and progression of a number of comorbid diseases, which additionally worsen the course of COPD and negatively affect the prognosis [8,9,10,11]. In clinical practice, the combined course of COPD and pulmonary tuberculosis is often observed, which leads to a more severe clinical picture of the combined conditions, an increased risk of complications, and reduced effectiveness of standard therapeutic approaches in the management of this category of patients [12]. Both diseases share many common links, including risk factors such as smoking and low socioeconomic status, as well as impaired lung structure and function. A history of tuberculosis is a risk factor for the development of COPD. In a large analysis of multinational population-based studies, the adjusted odds of developing COPD were 3.78 times higher in participants with a history of tuberculosis than in those without a history of tuberculosis [13]. This is because post-tuberculosis syndrome is associated with the development of airway obstruction and respiratory symptoms [14,15]. In the multicenter, cross-sectional, population-based Burden of Obstructive Lung Disease study, a history of tuberculosis was associated with both airway obstruction and decreased spirometric indices [16]. The study in miners showed decreased lung function after diagnosis of tuberculosis, especially in the first six months. Tuberculosis recurrences further reduced lung function: after one, two, and three or more episodes of tuberculosis, the decreases in FEV1 (forced expiratory volume in one second) were 153 mL, 326 mL, and 410 mL, respectively [17]. Importantly, patients who have completed treatment for severe pulmonary tuberculosis often remain severely impaired in terms of respiratory function. In a study including 76 patients, improvement of lung function was observed in only 54% of patients, while 28% and 24% of patients, respectively, had residual airflow limitations or a restrictive type of respiratory impairment. The degree of lung infiltration on radiologic examination both at baseline and after chemotherapy was significantly associated with FEV1 (% of baseline) [18]. Importantly, patients who have completed treatment for severe pulmonary tuberculosis often still exhibit severe respiratory impairment. A history of tuberculosis has also been shown to play an important role in the natural course of COPD. In patients with a history of tuberculosis, COPD was diagnosed 4 years earlier, and patients had more hospitalizations and died 5 years earlier compared with patients without tuberculosis [19]. Furthermore, patients with chronic airflow obstruction due to tuberculous lung disease, in addition to lower pulmonary function scores, had a worse response to bronchodilators (27% vs. 82%) than patients with COPD alone [20].

The problem of the comorbid course of COPD and tuberculosis also includes other clinical aspects. There are complexities and limitations in drug therapy. Severe COPD, for example, in some clinical situations involves the prescription of inhaled corticosteroids, which may have adverse effects on concomitant tuberculosis. An important issue relates to infectious exacerbations of COPD, their impact on tuberculosis, and the choice of effective antibacterial agents. In addition, there is increasing evidence that COPD has extrapulmonary clinical manifestations such as hypotrophy and peripheral muscle weakness, comorbid atherosclerosis, and diabetes mellitus, disturbances in the composition of the gut microbiota, etc. [10,21,22,23]. These same factors are also an important issue in tuberculosis, which further enhances the understanding of the complexity of the links between these diseases when comorbid.

In this regard, in addition to analyzing the clinical characteristics of comorbidity, it seems relevant to identify its fundamentals. Pathophysiologic characteristics of COPD and tuberculosis comorbidity include cross-immune relationships that determine the development and progression of both diseases. These mechanisms are based on the contribution of innate and adaptive immunity, cytokine and chemokine pathways, as well as features of intercellular interactions at the tissue and molecular levels [9,24,25].

**Figure 1:**
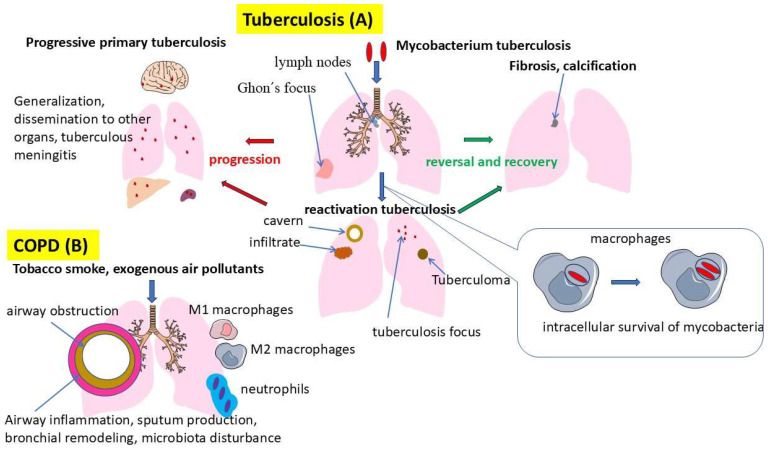
Lung tuberculosis (**A**) and COPD (**B**), having some common risk factors and mechanisms of development associated with inflammation, form a separate clinical phenotype in which the polymorphism of morphologic, radiologic, and clinical manifestations of tuberculosis plays an important role due to variability in immune response, structural changes in lung tissue, and individual features of disease pathogenesis. Note: A. Respiratory tuberculosis is an infectious disease caused by Mycobacterium tuberculosis. The main morphological manifestation of tuberculosis is the formation of specific granulomas with a characteristic histological structure: the presence of central caseous necrosis surrounded by a shaft of epithelioid cells, macrophages, lymphocytes, and giant Langhans cells, which reflects a cell-mediated immune response to the penetration of the pathogen. At first exposure to mycobacteria, more often in childhood, primary tuberculosis develops, which manifests itself by the formation of a primary affect (Ghon complex) in the lungs. The primary affect is characterized by an area of caseous tissue degeneration in the area of initial pathogen inoculation (predominantly subpleurally in the lower/middle lobes), tuberculous lymphangitis with caseous lymphadenitis of regional nodes, and often reactive pleurisy. The course of primary tuberculosis varies from asymptomatic with subsequent self-healing and calcification of the focus to manifest progressive tuberculosis with the risk of dissemination, development of serious complications and tuberculous meningitis. Secondary tuberculosis develops due to reactivation of latent tuberculosis infection or exogenous MBT superinfection and is characterized by polymorphism of morphological forms, localized mainly in the apical parts of the lungs. Focal tuberculosis is usually represented by single dense fibrous foci (up to 10 mm) within 1–2 segments with minimal perifocal inflammation and a clinically asymptomatic course. In contrast, infiltrative tuberculosis is characterized by predominantly exudative-necrotic changes with extensive areas of caseification surrounded by inflammatory infiltration, with a tendency to destruction of lung tissue and formation of decay cavities. In disseminated tuberculosis, there is hematogenous or lymphogenous spread of the pathogen with the formation of multiple foci and foci of infiltrative character. Tuberculoma is a form of secondary tuberculosis, which is an encapsulated focus of caseosis ranging in size from 1–2 to 5 cm or more, usually clinically silent and often detected during prophylactic radiologic examinations. Fibrotic cavernous tuberculosis is the final stage of development of all forms of respiratory tuberculosis with the formation of thick-walled cavities (caverns) with a three-layer wall (pyogenic, granulation, fibrous zones), which are surrounded by massive pericavitary sclerosis, bronchial deformation and destruction of parenchyma. In fibrotic cavernous tuberculosis, there is a high risk of multidrug-resistant and extensively drug-resistant MBT. B. Chronic obstructive pulmonary disease (COPD) is associated with chronic exposure to inhaled particles and gases, leading to the development of inflammation in the bronchi involving various cells. Prolonged inflammation leads to symptoms, development of bronchial obstruction, and emphysema. Neutrophils and macrophages are involved in bronchial remodeling through the production of elastases, matrix metalloproteinases. Disturbances in pro- and anti-inflammatory polarization of macrophages leads to chronicization of inflammation and development of systemic inflammation.

COPD is thought to develop as a result of prolonged exposure to harmful inhalation agents, predominantly including tobacco smoke and air pollutants (Figure 1). This exposure results in chronic inflammation of the airways and lung parenchyma, involving various types of immune system cells such as neutrophils, macrophages, and T lymphocytes. In this case, the migration and activation of excessive numbers of immune cells causing inflammation is promoted by increased expression of pro-inflammatory cytokines and chemokines, which initiates and enhances tissue damage. Chronic inflammation leads to airway remodeling and the destruction of alveolar structures through various mechanisms, which clinically manifests as airway obstruction and emphysema [26,27]. Transforming growth factor-β (TGF-β) and epidermal growth factor (EGF) play an important role in airway remodeling and are key mediators secreted by epithelial cells and macrophages. TGF-β and EGF stimulate fibroblast proliferation and extracellular matrix production, leading to airway wall thickening and fibrosis [28].

In turn, pulmonary tuberculosis caused by *Mycobacterium tuberculosis* is also characterized by complex interactions between the mycobacteria and the host immune system (Figure 1). *M. tuberculosis* has many tools that allow it to adapt to human immune defenses. The bacterium has a multilayered cell wall, the center of which is a complex of mycolic acids, arabinogalactan, and peptidoglycan [29]. Arabinogalactan performs mainly a structural function, “binding” peptidoglycan and mycolic acids, thus forming a rigid and hydrophobic barrier of mycobacteria [30,31]. These structures impede the entry of many hydrophilic antibiotics and antiseptics, providing high resistance of the pathogen in the external environment and in the host organisms [32]. In turn, an important role in the adaptation of *M. tuberculosis* to intracellular existence is played by lipoarabinomannan and other lipid components, which are able to modulate the immune response by suppressing the production of a number of cytokines and inhibiting the fusion of phago- and lysosomes, thus preventing the full activation of macrophages [33]. Lipoarabinomannan mainly inhibits the production of key pro-inflammatory cytokines such as TNF-α and IL-12, and may enhance the production of the anti-inflammatory cytokine IL-10 [34]. After infection, mycobacteria are phagocytized by alveolar macrophages, but are able to avoid intracellular elimination through various adaptation mechanisms [35]. This contributes to the prolonged persistence of immune response activity and the formation of granulomas, structures composed predominantly of epithelioid cells, Langhans giant cells, and lymphocytes. Granulomatous inflammation develops as a consequence of the body’s attempt to localize the infection. In this case, the prolonged immune response of the host contributes to damage to the lung tissue and the development of destructive changes, known as caverns [36]. An additional defense is the ability of the pathogen to persist for a long time in granulomas, where low oxygen levels, an acidic environment, and a lack of nutrients force the bacteria to change into L-forms (“dormant” *M. tuberculosis*), resistant to the effects of both endogenous immune mechanisms and many anti-tuberculosis drugs [37].

In COPD and tuberculosis comorbidity, chronic inflammation with mucus hyperproduction and bronchial remodeling contributes to easier penetration and persistence of mycobacteria due to loss of natural barriers in the form of turbulent air movement in the airways, decreased mucociliary clearance, production of secretory humoral defense factors (IgA, lysozyme, defensins, and surfactant) [38]. Disturbed function of alveolar macrophages and decreased local immunity in patients with COPD create favorable conditions for the latent course of tuberculosis and the risk of its reactivation [39].

Despite significant progress in understanding the clinical characteristics of COPD and pulmonary tuberculosis, substantial gaps remain regarding the mechanisms by which persistent Mycobacterium tuberculosis infection may potentiate or accelerate the inflammatory response characteristics of COPD. The inflammatory process is a fundamental pathophysiologic mechanism underlying both COPD and pulmonary tuberculosis. Both diseases are characterized by complex interactions between pathogens, immune system cells, and respiratory system tissues, resulting in progressive impairment of respiratory function [40]. Moreover, the levels of inflammatory indices were higher in patients with COPD and tuberculosis compared to patients without this comorbidity [41]. Thus, the study of COPD and tuberculosis comorbidity has led to the realization that these diseases, when coexisting together, can form the phenotype of tuberculosis-associated obstructive pulmonary disease (TOPD). In addition to this phenotype, which is based on clinical data, the biological basis of this comorbidity corresponds to the tuberculosis-associated COPD endotype [42,43].

Therefore, the aim of the current review is to discuss the common immune mechanisms underlying COPD and pulmonary tuberculosis. In addition, the review includes an analysis of the role of individual cells and mechanisms of the innate and adaptive immune system in the pathogenesis of COPD and tuberculosis alone and in comorbid courses.

## 2. Cytokines and Chemokines in the Pathogenesis of COPD and Tuberculosis Comorbidity: The Role of Inflammatory Mediators and Their Clinical Significance

The pathophysiological relationship between COPD and tuberculosis is due to the intersection of key inflammatory and immune pathways of disease pathogenesis. Both diseases are characterized by chronic inflammation involving innate and adaptive immunity, dysregulation of cytokine balance, and increased activation of cellular mediators of inflammation [44]. Inflammatory mediators play an important role in the development of COPD and tuberculosis, as they trigger and support the immune response. Among them are cytokines and chemokines involved in the formation of the systemic inflammatory process that determines pulmonary and extrapulmonary manifestations of the diseases. The key cytokines and chemokines that play a role in the comorbidity of COPD and tuberculosis are interleukins 1β, 6, and 8 (IL-1β, IL-6, IL-8), TNF-α, and interferon gamma (IFN-γ) [45]. IL-1β is a key pro-inflammatory cytokine secreted predominantly by activated macrophages and peripheral blood monocytes. It plays a central role in triggering and maintaining the inflammatory response in both diseases. In COPD, IL-1β promotes neutrophil migration into the airways, enhances mucus production, and stimulates airway remodeling [46]. Elevated levels of IL-1β correlate with the severity of obstruction and the frequency of exacerbations. It can also induce apoptosis of epithelial cells, contributing to the destruction of lung tissue [46,47]. In turn, in tuberculosis, IL-1β is involved in granuloma formation, thereby limiting the spread of *M. tuberculosis* in tissues [48]. However, excessive IL-1β secretion can lead to caseous necrosis, destruction of lung tissue, and worsening of the clinical course of the disease [49]. In comorbidity, a synergistic increase in IL-1β levels may exacerbate the inflammatory process, leading to more severe damage to the pulmonary parenchyma and worsening of respiratory function.

Another interleukin, IL-6, is a multifunctional cytokine involved in the regulation of immune response, inflammation, and hematopoiesis [50]. In COPD, IL-6 contributes to systemic inflammation and is associated with systemic manifestations of the disease, including weight loss and other systemic manifestations of the disease [51]. It is also involved in B-cell activation and T-cell differentiation, affecting the adaptive immune response [52,53]. Elevated IL-6 levels in tuberculosis are correlated with the active period of the disease and may reflect the degree of bacterial load. IL-6 is involved in the initiation of the acute phase of inflammation and stimulates the production of acute phase proteins in the liver [54]. In comorbidity, IL-6 may increase systemic inflammation, contributing to associated metabolic disorders and worsening the patient’s overall condition.

Interleukin 8 (IL-8) is a potent chemoattractant for neutrophils and plays an important role in their activation and migration [55]. In COPD, increased secretion of IL-8 in the airways leads to an accumulation of neutrophils, which, by releasing proteases and reactive oxygen species, contribute to lung tissue damage and airway obstruction [56]. In addition, chronic inflammation in COPD leads to excessive mucus production due to hyperplasia of goblet cells and submucosal glands. This occurs under the influence of inflammatory mediators such as IL-8 and TNF-α, which stimulate gene expression and mucus secretion [57,58]. In tuberculosis, IL-8 is involved in the formation of an inflammatory infiltrate around infected macrophages, which delineates specific inflammation from surrounding tissues and contributes to infection control by preventing the spread of the zone of caseous necrosis [59]. However, excessive neutrophil activation can in turn also lead to tissue damage and lysis of lung tissue [60]. In comorbidity, the synergistic effect of increased IL-8 production may increase neutrophilic inflammation, accelerating the progression of both diseases.

TNF-α is also one of the main mediators of systemic inflammation, which in turn also plays an important role in the immune response to various infections [61]. In COPD, TNF-α promotes neutrophil and macrophage activation, increases vascular permeability and stimulates tissue remodeling. It is associated with systemic manifestations of COPD and may contribute to the development of resistance to corticosteroid therapy [62]. In tuberculosis, TNF-α is essential for granuloma formation, limiting *M. tuberculosis* growth and multiplication. TNF-α deficiency is associated with dissemination of tuberculosis infection, but its overproduction can in turn lead to tissue necrosis [63]. In comorbidity, elevated TNF-α levels may exacerbate inflammation, contributing to a more severe disease course and increasing the risk of complications [64]. TNF-α is also known as “cachexin” due to its involvement in the development of hypotrophy and weakness of skeletal muscles, mainly of the lower limbs, which is an important complication of COPD that has received more attention in recent years. Indeed, physical weakness is considered as one of the phenotypes of COPD and significantly affects quality of life and prognosis.

In the immune response against intracellular pathogens, IFN-γ is a key cytokine, modulating the immune response, particularly in tuberculosis. In it, IFN-γ is critical for macrophage activation and killing of *M. tuberculosis* by completed phagocytosis [65]. IFN-γ deficiency leads to increased susceptibility to tuberculosis infection. In turn, in COPD, the role of IFN-γ is less pronounced, but it may participate in the chronicization of inflammation and activation of the macrophage component of inflammation [66]. In comorbidity, IFN-γ imbalance may lead to ineffective infection control and increased inflammation, affecting the outcome of both diseases.

Thus, both COPD and tuberculosis are characterized by disturbances in the production of cytokines and chemokines, which is part of their natural course. It is important to note that inflammation in TOPD has its own peculiarities. Patients with tuberculosis-associated COPD (T-COPD) were found to have higher levels of inflammatory markers such as IL-6, C-reactive protein, and NLR than patients with smoking-associated COPD [25]. Another study showed higher levels of inflammatory markers (systemic inflammatory response index (SIRI), C-reactive protein-to-lymphocyte ratio (CLR), and eosinophil count-to-lymphocyte count ratio (ELR)) in patients with TOPD than in patients with COPD [41].

Thus, key cytokines and chemokines such as IL-1β, IL-6, IL-8, TNF-α and IFN-γ play an important role in the pathogenesis of both COPD and tuberculosis. Their interaction in comorbidity enhances the inflammatory response, contributes to lung tissue damage, and worsens clinical outcomes. An imbalanced cytokine profile may impair the effective immune response to *M. tuberculosis*, increasing the risk of tuberculosis progression in COPD patients. In this regard, cytokines represent potential targets for drug therapy. Modulation of their activity may improve clinical outcomes and slow disease progression [67]. Levels of these cytokines can be used to assess inflammatory activity, predict disease course, and monitor the efficacy of therapy [68,69]. However, their therapeutic potential in COPD and tuberculosis is limited. Studies on the use of known monoclonal antibodies against IL-1β, TNF-α, and IL-6 did not show their effectiveness or had negative side effects [70,71] (Table 1). However, recent recommendations for the treatment of COPD have included an IL-4 and IL-13 receptor antagonist for type 2 inflammation [72].

Interleukin antagonists are also considered as potential means of modulating the immune response in respiratory tuberculosis, but their effect on the course of infection remains ambiguous [79,80,81]. On the one hand, blockade of proinflammatory cytokines (in particular, IL-1β and IL-6) can reduce the excessive activity of macrophages and neutrophils, decrease the production of mediators responsible for hyperinflammatory states, and thus prevent the destruction of lung tissue [82,83]. It is believed that it is necrosis and cavernous formation that largely determine the severity of the course of tuberculosis [84], so limiting excessive inflammation can theoretically lead to a more favorable outcome of the disease. On the other hand, IL-1β and IL-6 participate in the formation of tuberculosis granulomas [85,86], regulate the activation of macrophages and T-lymphocytes, and influence the secretion of interferon-γ and other key cytokines [87,88,89]. As a consequence, excessive blockade of IL-1β (e.g., canakinumab) or IL-6 (tocilizumab, sirukumab, siltuximab, olokizumab, and clazakizumab) can lead to weakening of antimycobacterial immunity, which increases the risk of accelerated multiplication of mycobacteria and reactivation of latent infection [90]. At the same time, modulation of IL-27 from the IL-12 family of cytokines is considered a promising direction for immunotherapy in respiratory tuberculosis. IL-27 has a dual action: on the one hand, it enhances Th1-response due to stimulation of interferon-γ production, which is important for suppression of *M. tuberculosis* population growth, and on the other hand, it is able to restrain hyperinflammatory states by inhibiting Th17 cells and blocking excessive release of proinflammatory cytokines [91]. Overall, at present, the evidence on the effects of interleukin antagonists on tuberculosis is mainly represented by observational studies and case descriptions in patients who have used interleukin antagonists to treat autoimmune or inflammatory diseases [92]. Such studies have repeatedly documented cases of latent tuberculosis progressing to clinically manifested disease due to a decreased anti-tuberculosis response [93]. This has led to the need for routine *M. tuberculosis* screening (Mantoux or IGRA tests) before starting therapy with biologic drugs [94]. At the same time, patients who received prophylactic treatment (usually including isoniazid) when latent tuberculosis was detected had a reduced risk of developing active tuberculosis [95]. It has been shown in experimental models that complete long-term blockade of IL-1β or IL-6 can disrupt the structure of tuberculous granulomas and weaken the control of the mycobacterial population, whereas time-limited blockade of these cytokines at early stages of hyperinflammation in some cases showed potential benefit in preventing severe, often destructive, tissue damage [83,96].

In this regard, the described cytokines, although promising therapeutic targets, at the current time still require new studies to evaluate their therapeutic potential.

## 3. Mechanisms of Cellular Inflammation in COPD and Tuberculosis Co-Morbidity: Contribution of Neutrophils, Macrophages and Dendritic Cells

The combined course of chronic obstructive pulmonary disease (COPD) and tuberculosis involves complex interactions between different cells of the immune system as well as pathogens, leading to increased cellular inflammation. Chronic inflammation in COPD is known to lead to disruption of the structure of the airway microbiome, which in healthy individuals is maintained through complex interactions between the normal microbiota and immune cells [97,98]. The respiratory microbiome is represented by many species of bacteria and fungi that exhibit diverse, largely unknown functions. Disruption of the structure of the airway microbiota contributes to exacerbations of COPD, which have a negative impact on disease progression and prognosis [99,100,101,102]. In this regard, the involvement of various immune cells in inflammation in COPD and TOPD is of considerable clinical and research interest. It is known that the cellular component of inflammation in COPD and tuberculosis comorbidity is represented by various cells, mainly neutrophils, macrophages (monocytes), and dendritic cells [103].

### 3.1. The Role of Neutrophils

Neutrophils, being the key effector cells of innate immunity, play a central role in the pathogenesis of inflammatory processes in COPD and pulmonary tuberculosis [104]. Their activation and functional activity determine both protective and pathological effects that influence the course and outcome of these diseases. Neutrophils have a wide range of defense mechanisms that allow them to effectively respond to infectious agents and modulate the inflammatory response. They are able to capture and destroy microorganisms through oxygen-dependent (formation of reactive oxygen species) and oxygen-independent (lysosomal enzymes) mechanisms [105]. At the same time, the process of lysosome degranulation itself plays an important role via the release of granules containing proteolytic enzymes (neutrophil elastase, cathepsins, matrix metalloproteinases) and antimicrobial peptides (defensins, lactoferrin), which leads both to the destruction of pathogens and, in some situations, tissue damage [106]. Additionally, one of the mechanisms of infectious defense in neutrophils is the formation of neutrophil extracellular traps (NETs), structures that are DNA networks bound to proteases. These traps aim to capture and neutralize microorganisms, preventing their spread [107]. Adhesion and diapedesis of neutrophils across the vascular wall is promoted by increased expression of selectins and integrins on endotheliocytes and neutrophils [108]. The stimulation of pro-inflammatory activity of neutrophils is promoted by increased expression of IL-8 (CXCL8), GRO-alpha (Growth-Regulated Oncogene-alpha or CXCL1), and leukotriene B4 (LTB4), which stimulate them via CXCR1 and CXCR2 receptors [109]. In doing so, TNF-α and IL-1β enhance neutrophil activation, increasing their survival and functional activity [109,110].

A characteristic feature of COPD is chronic neutrophilic inflammation, which contributes significantly to the progression of the disease and the development of exacerbations. As already indicated, one of the key functions of neutrophils in this context is the production of cytokines and chemokines [111]. Neutrophils synthesize pro-inflammatory mediators (IL-8, TNF-α), increasing chemotaxis and activation of other cells of the immune system. Under the influence of chemokines (e.g., IL-8, LTB4), neutrophils migrate to the airways and pulmonary parenchyma [112]. Pro-inflammatory cytokines (TNF-α, IL-1β) and tobacco smoke promote neutrophil activation, enhancing their ability to phagocytose and degranulate [113]. Released proteases and reactive oxygen species lead to destruction of the extracellular matrix, breakdown of elastic fibers, and airway remodeling, contributing to the development of emphysema [114]. Increased production of reactive oxygen species causes damage to cellular structures, increasing inflammation and impairing antioxidant defenses [115].

In tuberculosis infection, neutrophils have a dual role, participating both in the defense of the organism and in pathological processes. Neutrophils play a protective role in the earliest stages of tuberculosis infection. They phagocytize *M. tuberculosis* in an attempt to limit the spread of infection. Further, through the release of reactive oxygen species and antimicrobial peptides, neutrophils participate in the destruction of mycobacteria [116]. Neutrophils may be involved in the structure of granulomas, affecting their stability and functionality [117]. However, excessive activation of neutrophils leads to the release of proteases and reactive oxygen species, promoting the spread of caseous necrosis and the formation of lung tissue destruction [118]. The combination of COPD and tuberculosis leads to an increased neutrophil component in inflammation, which aggravates pathologic changes in the lungs. The combined action of chemokines and proinflammatory cytokines in both diseases increases neutrophil migration and activation in lung tissue [119]. At the same time, the combination of protease activity and oxidative stress increases the destruction of parenchyma, accelerating the development of fibrosis, emphysema progression and cavern formation [120]. Chronic neutrophilic inflammation can suppress adaptive immunity, reducing the effectiveness of the anti-tuberculosis response and increasing the risk of spread of infection, leading to an increased frequency of exacerbations, decreased respiratory function, and increased mortality.

### 3.2. The Role of Macrophages

**Figure 2:**
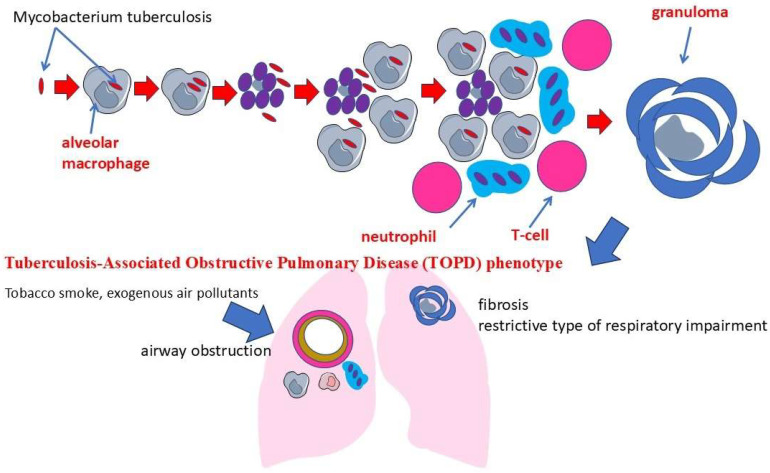
Macrophages play an important role in the pathogenesis of both COPD and tuberculosis.

Macrophages, like neutrophils, are key cells of innate immunity and play a central role in the pathogenesis of inflammatory processes in the comorbidity of chronic obstructive pulmonary disease and pulmonary tuberculosis (Table 2) (Figure 2).

They are involved in pathogen recognition, phagocytosis, antigen presentation, and regulation of the immune response through the secretion of a wide range of cytokines and chemokines [137]. In COPD, chronic exposure to inhaled pollutants such as tobacco smoke and industrial emissions leads to activation of alveolar macrophages [138]. These activated macrophages secrete proinflammatory cytokines including IL-1β, TNF-α, and IL-6, which enhance the inflammatory response and attract additional immune system cells to the airways [139]. In addition, they produce reactive oxygen species and proteolytic enzymes, contributing to oxidative stress and lung tissue destruction. This leads to airway remodeling, emphysema, and progression of obstructive changes [140]. In the context of tuberculosis infection, macrophages are the first cells to interact with *M. tuberculosis*. After phagocytosis of mycobacteria, macrophages, like neutrophils, attempt to destroy them through oxygen-dependent and oxygen-independent mechanisms. However, *M. tuberculosis* has the ability to avoid eradication by inhibiting phagosome maturation and suppressing the antibacterial responses of macrophages [141]. This leads to persistence of infection and chronicization of the inflammatory process [142]. It is primarily macrophages that are involved in the formation of granulomas, structures aimed at limiting the spread of bacteria, but that also contribute to tissue damage when over-activated (Figure 2) [143]. The persistence of solid granulomas in infected individuals is a sign of a balanced opposition between *M. tuberculosis* and the host, and any imbalance can lead to the development of disease. However, when immunity is compromised, *M. tuberculosis* becomes activated and begins to multiply. This leads to necrosis of infected macrophages and release of intracellular bacteria that can infect new cells and spread to other tissues [144].

An important factor determining the functional activity of macrophages is their polarization into M1 and M2 phenotypes due to immunometabolic reprogramming [145]. Macrophages of the M1 phenotype, or “classically activated” macrophages, are characterized by pronounced proinflammatory properties. They produce high levels of proinflammatory cytokines (TNF-α, IL-1β, IL-6), reactive nitrogen species (NO) and reactive oxygen species (ROS), enhancing the inflammatory response and participating in the destruction of pathogens. M1-macrophages contribute to the activation of the Th1 response, associated with the activation of type 1 T-helper cells, and enhance cellular immunity. In contrast, M2-phenotype macrophages, or “alternatively activated” macrophages, have anti-inflammatory and reparative properties. They secrete anti-inflammatory cytokines such as interleukin-10 (IL-10) and transforming growth factor beta (TGF-β), and promote tissue regeneration, remodeling, and angiogenesis. M2-macrophages are involved in suppressing excessive inflammation and maintaining homeostasis, as well as stimulating the humoral immune response [146,147,148,149].

In the comorbidity of COPD and pulmonary tuberculosis, there is a complex imbalance between M1 and M2 macrophage polarization, which significantly affects the course and outcome of each disease. Chronic inflammation in COPD favors a predominance of M1-macrophages, enhancing the inflammatory response and lung tissue damage [150]. This creates favorable conditions for *M. tuberculosis* persistence and impairs infection control. The predominance of M1-macrophages increases tissue damage, oxidative stress, and protease activity, contributing to the progression of COPD and the destruction of pulmonary parenchyma in tuberculosis. On the other hand, *M. tuberculosis* can induce M2-polarization of macrophages through the secretion of mycobacterial lipoproteins and other virulence factors, which helps bacteria to evade immune control and persist in the body [151]. Insufficient M2-macrophage activity leads to decreased reparative processes, impaired tissue remodeling, and chronicization of inflammation. Thus, M1 macrophages contribute to the suppression of tuberculosis progression. However, uncontrolled inflammation induced by M1 macrophages can lead to severe tissue damage. In this regard, immunosuppressive regulation of M2 macrophages and Th2 cells plays an important role [144,152].

Macrophage polarization is regulated by multiple factors including microenvironmental cytokines, pathogen interactions, and metabolic pathways. IFN-γ and lipopolysaccharides promote M1 polarization, whereas interleukin-4 (IL-4) and interleukin-13 (IL-13) stimulate the M2 phenotype [153]. In comorbidity, the cytokine profile changes, which affects macrophage polarization and the nature of the immune response [154]. Understanding the mechanisms of macrophage polarization and its role in the pathogenesis of comorbidity is important for the development of new therapeutic strategies. Modulation of macrophage polarization can be achieved by targeting signaling pathways such as JAK/STAT, NF-κB, and MAPK [155]. This may help to restore the balance between pro- and anti-inflammatory processes, reduce chronic inflammation, improve pathogen elimination, and stimulate regenerative processes in the lung.

Polarization of macrophages into M1 and M2 phenotypes is accomplished by switching their metabolism. While M2 macrophages are characterized by normal glycolysis and oxidative phosphorylation activity, M1 macrophages rely predominantly on glycolysis for energy, and the tricarboxylic acid cycle and oxidative phosphorylation are impaired [156]. Interestingly, M. tuberculosis can affect the metabolic activity of macrophages, which may contribute to mycobacterial survival through several mechanisms [157,158,159]. In this regard, macrophages are promising targets for therapies aimed at reducing the resistance of *M. tuberculosis* to intracellular elimination [160]. Stimulation of autophagy and enhancement of antibacterial mechanisms in macrophages may improve the efficacy of anti-tuberculosis therapy.

### 3.3. The Role of Dendritic Cells

Dendritic cells (DCs) play an important role in recognizing and responding to foreign agents, protecting the body and preventing excessive inflammatory reactions. Their function is particularly important in the lungs, which are constantly in contact with the external environment and at risk of infection. DCs are key professional antigen-presenting cells in the immune system, playing a central role in the initiation and regulation of the adaptive immune response. They perform antigen capture, processing, and presentation to T lymphocytes, thereby linking innate and adaptive immunity [161]. In the comorbid course of chronic obstructive pulmonary disease and pulmonary tuberculosis, the functional activity of DCs acquires special importance, influencing the nature of the immune response and the course of the diseases. DCs are present in various tissues of the body, including the respiratory tract and pulmonary parenchyma. In the respiratory tract, they are found in the epithelial layer and subepithelial tissue, where they monitor the environment for foreign antigens [162].

Upon ingestion of pathogenic microorganisms or inhalation stimuli, DCs capture antigens by phagocytosis, endocytosis, or macropinocytosis. After antigen capture, DCs migrate to regional lymph nodes, where they mature and present the antigen on the surface in complex with major histocompatibility complex molecules of class II for CD4+ T-lymphocytes and class I for CD8+ T-lymphocytes. The process of antigen presentation is accompanied by marked expression of costimulatory molecules (CD80, CD86, CD40) and secretion of cytokines such as interleukin-12 (IL-12), which are necessary for full activation of young T cells [163]. Activation of T lymphocytes leads to their proliferation and differentiation into effector cells, including Th1, Th2, Th17, and regulatory T cells (Treg), which direct the subsequent immune response [164]. In COPD, chronic inflammation and exposure to inhaled stimuli can alter the function and phenotype of DCs. Tobacco smoking and other harmful agents contribute to the decreased antigen-presenting capacity of DCs, impaired migration, and an altered cytokine secretion profile [165]. This can lead to an imbalance between pro- and anti-inflammatory reactions, contributing to the chronicization of inflammation and reduced effectiveness of the immune response to pathogens. In the case of tuberculosis infection, *M. tuberculosis* interacts with DCs, affecting their function. Mycobacteria can inhibit DC maturation, suppress the expression of costimulatory molecules, and alter cytokine secretion, leading to insufficient T-lymphocyte activation and persistence of infection [166]. In addition, *M. tuberculosis* can induce the expression of programmed cell death ligand 1 (PD-L1) on the surface of DCs, which contributes to the inhibition of the T-cell response through the PD-1/PD-L1 pathway [167].

In the comorbidity of COPD and tuberculosis, dendritic cell dysfunction is exacerbated, with significant clinical consequences. Disruption of antigen presentation and activation of adaptive immunity leads to insufficient elimination of *M. tuberculosis*, progression of infection and worsening control of the inflammatory process. Chronic inflammation in COPD contributes to further damage to DCs, reducing the effectiveness of immune control and increasing the risk of exacerbations and complications. The polarization of the T-cell response also depends on the cytokine environment created by DCs. Under normal conditions, DCs secreting IL-12 promote the differentiation of young CD4+ T lymphocytes into Th1 cells, which produce IFN-γ and activate macrophages to kill intracellular pathogens such as *M. tuberculosis* [168]. However, DC dysfunction decreases IL-12 production and increases IL-10 and TGF-β secretion, which promotes the differentiation of Treg and Th2 cells that suppress cellular immunity and contribute to the chronicity of infection [169]. In addition, DCs are involved in the formation of immunologic tolerance and the maintenance of airway homeostasis [170]. In COPD, impairment of this function may lead to the development of autoimmune reactions and hypersensitivity to infections. DCs also interact with other cells of the immune system, including B-lymphocytes and natural killer cells (NK cells), modulating the overall immune response [171].

Thus, when COPD and pulmonary tuberculosis are combined, neutrophils, macrophages, and dendritic cells form a pathogenetically closely intertwined network that determines the intensity and orientation of the inflammatory process in tissues. On the one hand, neutrophils actively participate in the destruction of pathogens through the release of proteases, reactive oxygen species, and antimicrobial peptides, with their excessive activation increasing the destruction of extracellular matrix and the progression of obstructive changes. On the other hand, impaired macrophage polarization, leading to an imbalance of the M1- or M2-phenotypes of macrophages, which is regulated by the cytokine milieu, may support prolonged pathological inflammation and persistence of *M. tuberculosis*. At the same time, dendritic cells, which are the key antigen-presenting elements in the formation of immunity, actively modify the specific immune response, which in comorbidity conditions can also lead to impaired control of infection and increased tissue damage.

## 4. Innate Immunity in COPD and Tuberculosis: Activation of Toll-like and Nod-like Receptors and Their Influence on Inflammation

Disorders of innate immunity are an important part of pathogenesis in COPD and pulmonary tuberculosis and their comorbid course. Innate immunity is the first line of defense of the organism, providing a rapid and effective nonspecific response to the introduction of pathogens. The basis of its functioning is the recognition of conservative structural components of pathogens with the help of special receptors of the immune system: pattern recognition receptors [172]. Of particular importance among pattern recognition receptors are toll-like receptors (TLRs) and NOD-like receptors (NLRs), which initiate signaling pathways leading to the activation of the immune response and the production of proinflammatory cytokines [173].

TLR receptors are transmembrane proteins located on the surface of immune system cells or in endosomes, and the most studied in bacterial infections are their TLR2 and TLR4 variants. The TLR2 receptor recognizes cell wall components of Gram-positive bacteria such as lipoproteins and lipoteichoic acid, whereas TLR4 interacts with lipopolysaccharides of Gram-negative bacteria [173]. In the case of tuberculosis infection, *M. tuberculosis* possesses unique lipid components such as lipoarabinomannan that can be recognized simultaneously by both TLR2 and TLR4 [174]. Activation of these receptors on macrophages, DCs, and airway epithelial cells leads to translocation of the transcription factor NF-κB into the nucleus, which stimulates gene expression of pro-inflammatory cytokines including IL-1β, IL-6, and TNF-α [175]. In turn, in chronic obstructive pulmonary disease, chronic exposure to tobacco smoke and other inhalant stimuli causes persistent activation of innate immunity via TLRs [176]. This leads to chronic inflammation of the airways and pulmonary parenchyma involving neutrophils, macrophages, and lymphocytes. In this case, increased expression of TLR2 and TLR4 on airway cells contributes to the inflammatory response and tissue damage. In addition, TLR activation can lead to resistance to glucocorticosteroids, complicating therapy for COPD [177].

In turn, NLRs are cytoplasmic proteins that recognize intracellular patterns of pathogens or products of cell damage. NLRs (NOD2) recognize mycobacterium muramyl dipeptide, which triggers inflammasome formation, caspase-1 activation, and IL-1β production, enhancing the immune response to defend against intracellular pathogens. Mycobacterium tuberculosis, in turn, employs immune evasion mechanisms such as inhibition of phagosome-to-lysosome fusion, masking of PAMP, and suppression of signaling pathways, which promotes their survival within cells and attenuates the immune response. Among NOD-like receptors, NLRP3, which is a key component of the NLRP3 inflammasome, has received special focus [178]. Inflammasome NLRP3 is a multiprotein complex that, upon activation, leads to caspase-1 activation and subsequent secretion of the active forms of IL-1β and interleukin-18 (IL-18) [179]. Inflammasome activation can be induced by a variety of exogenous and endogenous stimuli, including asbestos crystals or silicate particles, ATP, reactive oxygen species, and bacterial cell components [180]. NLRP3 inflammasome activation and subsequent IL-1β secretion play dual roles in tuberculosis infection. As noted in the previous sections, IL-1β contributes to an effective immune response against *M. tuberculosis* by enhancing macrophage activation and stimulating the production of other pro-inflammatory cytokines [85]. On the other hand, excessive inflammasome activation and excessive IL-1β secretion can lead to pathologic inflammation and lung tissue damage, promoting disease progression and cavern formation [181].

In COPD and tuberculosis comorbidity, a complex interaction between TLR and NLR signaling pathways occurs, which enhances the inflammatory response and tissue damage. Acting through different mechanisms, for example, TLR2 and NOD2 interact to enhance cytokine production, which is critical for an effective immune response to *M. tuberculosis* [182]. Chronic activation of TLR2 and TLR4 in COPD creates an inflammatory microenvironment that may promote *M. tuberculosis* persistence and reduce the efficacy of the immune response [183]. Simultaneously, activation of the NLRP3 inflammasome by mycobacterial components and cellular stress products in COPD leads to enhanced IL-1β secretion, which increases inflammation and lung damage [184]. An additional factor is oxidative stress, which is often observed in COPD due to increased production of reactive oxygen species and nitrogen species. Oxidative stress can directly activate the NLRP3 inflammasome or enhance its activation in response to other stimuli [185]. This creates a vicious circle where inflammation and oxidative stress mutually reinforce each other, leading to disease progression. Understanding the role of TLRs and NLRs in the pathogenesis of COPD and tuberculosis comorbidity has important clinical implications. Targeting these receptors and their associated signaling pathways represents a promising approach to therapy. NLRP3 inflammasome inhibitors or IL-1β blockers can be used to reduce pathologic inflammation [186]. However, a balance between suppressing excessive inflammation and preserving an effective immune response against *M. tuberculosis* must be considered. In addition, polymorphisms of genes encoding TLR and NLR may influence susceptibility to the development of COPD and tuberculosis, as well as the severity of their course [187]. It should also be remembered that different genetic variations in TLR2 and TLR4 are associated with altered susceptibility to infections and response to therapy [188]. The study of these genetic factors may contribute to the development of personalized medicine and more accurate prediction of the risk of comorbidity.

Thus, in the comorbidity of COPD and pulmonary tuberculosis, the realization of complex participation of mechanisms of innate immune response is observed, in which TLR and NLR play an important role. Their constant stimulation by external pathogens and endogenous stimuli during tissue damage in conditions of chronic inflammation leads to hyperexpression of cytokines and activation of NLRP3 inflammasome. As a result, a pathogenic circle is formed in which TLRs and NLRs, interacting with oxidative stress factors and impaired airway barrier function, maintain active inflammation and damage to the pulmonary parenchyma. Prolonged stimulation of innate immunity not only reduces the efficacy of antitubercular control but also exacerbates obstructive changes, complicating therapy and worsening prognosis. These data underscore the clinical relevance of investigating the genetic variability of TLR and NLR and the need to develop strategies aimed at modulating these signaling pathways, thereby allowing the body to balance between suppressing overactive inflammation and maintaining an adequate immune response in comorbid pathology.

## 5. Adaptive Immunity in COPD and Tuberculosis: The Role of T-Lymphocytes, B-Lymphocytes and Their Interactions

Although innate immunity plays a critical role in the first line of defense, adaptive immunity in turn provides high specificity and long-term memory, which is particularly important in chronic and recurrent infections, while playing a key role in the pathogenesis and control of chronic obstructive pulmonary disease and tuberculosis, especially when they are comorbid [189]. The central regulators of the adaptive immune response are T-helper (Th) cells, which direct it towards cellular or humoral immunity depending on the signals of the microenvironment [190]. In this case, the effectiveness of the anti-infection response and the degree of inflammation in lung tissue is determined by the balance between the subpopulations of T lymphocytes: Th1, Th2, and Th17. Th1 cells produce IFN-γ and stimulate cellular immunity by activating macrophages to kill intracellular pathogens such as *M. tuberculosis* [191]. Therefore, the Th1 response is particularly important for controlling and limiting the spread of mycobacteria in tuberculosis infection. However, in COPD, chronic inflammation and exposure to inhalation stimuli may impair Th1 cell differentiation, reducing the effectiveness of cellular immunity and promoting persistence of infection [192]. Another subpopulation, Th17 cells, produce interleukin-17 and participate in chemotaxis and migration of neutrophils to the site of inflammation. They play a dual role: on the one hand, they contribute to the defense against extracellular bacteria and fungi; on the other hand, they participate in the development of autoimmune and inflammatory diseases [193]. In COPD, increased activity of Th17 cells may increase neutrophilic inflammation, contributing to the destruction of lung tissue [194]. Accordingly, when comorbid with tuberculosis, an imbalance toward the Th17 response may lead to marked inflammation and tissue damage, worsening clinical outcomes. The third subpopulation, Th2 cells, also produce cytokines such as IL-4, interleukin-5, and IL-13, and direct the immune response towards humoral immunity and allergic reactions [195]. In COPD, the predominance of the Th2 response is less characteristic, but some studies indicate its possible involvement in the pathogenesis of the disease, especially in nonsmoking patients [196]. In turn, in tuberculosis, the predominance of the Th2 response may contribute to a decreased efficiency of cellular immunity and impaired control of infection [197]. The balance between Th1, Th17, and Th2 responses is very important to maintain an effective immune response without excessive tissue damage. Disruption of this balance in the comorbidity of COPD and tuberculosis may lead to inadequate infection control and the spread of pathologic, often destructive, inflammation.

In patients with COPD, especially during acute exacerbations, there is a significant decrease in the number of CD4+ T-cells, which leads to a decrease in the CD4+/CD8+ ratio [198,199]. In patients with tuberculosis, especially against the background of comorbidities, such as diabetes, there is a change in the frequency of CD4+ T-cell subtypes, including an increase in the number of central memory T-cells and a decrease in the number of naive and effector memory T-cells [200]. It should be emphasized that in tuberculosis, CD4+ T cells play a key role in the formation of an effective immune response. Additionally, increased expression of immune checkpoint molecules, such as BTLA, on CD4+ T cells is observed in patients with tuberculosis, which often correlates with disease progression [201]. Similar abnormalities are observed in the CD8+ T-lymphocyte population. Their number is also reduced in patients with COPD, which contributes to the general immune suppression observed in these individuals [198]. In the case of tuberculosis, CD8+ T cells demonstrate functional reorganization: in the chronic course of infection, they lose cytotoxic activity, switching to excessive cytokine production, which may contribute to MBT persistence [140]. In parallel, in such patients, the expression of proapoptotic molecules on CD8+ T cells increases, leading to their premature exhaustion and decreased efficiency of the immune response [141].

Other variants of T cells, regulatory T cells (Treg), are important modulators of the immune response, providing control over excessive inflammation and preventing autoimmune reactions. They, as well as M2-macrophages, produce anti-inflammatory cytokines such as IL-10 and TGF-β, suppressing the activation of effector T cells and the spread of inflammation [202]. In COPD, Treg function can be impaired, contributing to chronicity of inflammation and lung tissue damage [203]. In tuberculosis infection, Treg cells may limit excessive inflammation and tissue damage, but simultaneously promote *M. tuberculosis* persistence by suppressing the effector immune response [204]. In the context of comorbidity, Treg cell dysfunction may exacerbate pathologic inflammation in COPD and reduce the efficacy of anti-tuberculosis immunity. Therefore, an optimal ratio between effector T cells and Treg cells is necessary for effective control of infection and minimization of tissue damage.

When discussing the role of adaptive mechanisms of immunologic defense, one cannot avoid B-cells and humoral immunity, which play a significant role in the pathogenesis of COPD and tuberculosis. B cells differentiate into plasma cells that produce antibodies that are involved in pathogen neutralization and opsonization, facilitating phagocytosis [205]. In tuberculosis, specific antibodies may help to limit the spread of infection, although their protective role is less pronounced compared to cellular immunity [206]. In COPD, there is impaired B-cell function and an altered antibody production profile, which may contribute to increased susceptibility to infections and decreased efficiency of the immune response [207]. In addition, immune complex formation and complement activation may exacerbate inflammation and tissue damage [208]. Although opsonization of pathogens with antibodies improves their recognition and phagocytosis by macrophages and neutrophils, the efficiency of this process may decrease in chronic inflammation and impaired function of immune cells [209]. Genetic and epigenetic factors can influence the differentiation and function of T- and B-cells, which is reflected in the individual characteristics of the immune response and predisposition to the development of COPD and tuberculosis [210]. Polymorphisms of cytokine genes and their receptors can alter the balance between different lymphocyte subpopulations, affecting the course of diseases [211].

Thus, in COPD and pulmonary tuberculosis comorbidity, the adaptive immunity represented by different subpopulations of T-lymphocytes and B-cells forms a complex pathogenetic picture, in which the shift of the balance towards a certain phenotype of cellular immune response has a direct impact on the effectiveness of infection control and the prevalence of lung tissue damage. The predominance of Th1 cells contributes to more active elimination of *M. tuberculosis*, but this can be disturbed under the influence of chronic action of pollutants in COPD. An increase in the proportion of Th17 cells enhances neutrophilic inflammation and tissue damage, as does an imbalance toward a Th2 response or impaired function of Treg cells and B-cells, formation of immune complexes, and complement activation, which may in turn also reduce the effectiveness of antituberculosis defense and increase lung tissue decay. Such shifts do not mean changes in the genotype of immune cells, but reflect dynamic adaptation of their functional properties under the influence of the external factors, microenvironment, and signaling molecules present in comorbidity. As a result, a pathophysiological state is formed, in which insufficient control of infection is combined with chronicization of inflammation, remodeling of pulmonary parenchyma, and worsening prognosis in patients with this comorbid pathology.

## 6. Signaling Pathways in the Pathogenesis of COPD and Tuberculosis: Influence of NF-κB, MAPK and JAK/STAT on Inflammation and Tissue Damage

After analyzing the role of innate and adaptive immunity in the pathogenesis of COPD and pulmonary tuberculosis comorbidity, it is important to focus on the molecular mechanisms regulating the inflammatory process. Intracellular signaling pathways such as NF-κB, MAPK, and JAK/STAT are key factors linking the activation of cellular and humoral immunity to proinflammatory gene expression. These pathways regulate the expression of genes responsible for the production of pro-inflammatory cytokines, chemokines, and other inflammatory mediators, influencing the intensity and nature of the immune response and hence the course of diseases [212]. The NF-κB (nuclear factor kappa-light-chain-enhancer of activated B cells) mediated signaling pathway is one of the main transcription factors involved in the regulation of inflammatory processes in general. Under normal conditions, NF-κB is retained in the cytoplasm in an inactive state bound to IκB inhibitor proteins [213]. When exposed to pro-inflammatory stimuli such as cytokines (among them, e.g., TNF-α, IL-1β), bacterial components (*M. tuberculosis*), and reactive oxygen and nitrogen species, IκB kinase (IKK) is activated. IKK phosphorylates IκB, leading to its ubiquitination and subsequent degradation in the proteasome. The released NF-κB translocates to the nucleus where it binds to the promoters of target genes and stimulates their transcription. This includes genes for pro-inflammatory cytokines (IL-6, IL-8, TNF-α), adhesion molecules (ICAM-1, VCAM-1), enzymes involved in prostaglandin synthesis (COX-2), and other inflammatory mediators [214]. In COPD and tuberculosis comorbidity, NF-κB activation is enhanced due to the synergistic effect of chronic inflammation induced by inhalation stimuli in COPD and mycobacterial persistence in tuberculosis. This leads to hyperproduction of proinflammatory cytokines, increased migration and activation of immune cells in the lungs, increased oxidative stress and, as a consequence, to progressive damage of lung tissue [215]. Inhibition of NF-κB can lead to increased apoptosis and autophagy in infected macrophages, reducing the viability of intracellular *M. tuberculosis*. This suggests that NF-κB inhibitors may enhance the body’s ability to control infection [216].

Another important signaling pathway is mitogen-activated protein kinase (MAPK) cascades. This signaling pathway is involved in the transmission of signals from the cell surface to the nucleus in response to various stress stimuli. MAPK cascades include three major components: ERK (extracellular signal-regulated kinase), JNK (c-Jun N-terminal kinase), and p38 MAPK [217]. ERK is normally activated in response to growth factors and mitogens, participating in the regulation of cell proliferation and differentiation [218]. JNK and p38 MAPK are activated by stressors such as cytokines, UV radiation, oxidative stress, and bacterial cell components [219]. Activation of these kinases occurs through sequential phosphorylation in the MAPKKKK → MAPKKK → MAPK cascade. Activated ERK, JNK, and p38 phosphorylate various transcription factors, including AP-1 (c-Fos/c-Jun complex), ATF-2 and Elk-1, leading to the altered expression of genes associated with inflammation, apoptosis, and stress response [220]. In COPD and tuberculosis, activation of MAPK cascades contributes to an enhanced inflammatory response, and the increased production of pro-inflammatory cytokines and matrix metalloproteinases involved in lung tissue remodeling and destruction [221]. In turn, ERK plays a role in cell proliferation and preservation, but in comorbidity, its activation may promote smooth muscle cell and fibroblast hyperplasia in the airways, exacerbating obstruction [222]. JNK and p38 MAPK are involved in the regulation of apoptosis and cytokine production. JNK activation leads to phosphorylation of the transcription factor c-Jun, which is part of AP-1 and stimulates the expression of proinflammatory genes; p38 MAPK regulates the synthesis and mRNA stability of cytokines such as TNF-α and IL-1β, enhancing the inflammatory response [220]. Another key mechanism of signaling from cytokine and growth factors to the cell nucleus is the JAK/STAT signaling pathway [223]. This pathway involves the binding of cytokines (e.g., interferons, IL-6, IL-10) to their receptors, leading to their dimerization and activation of associated Janus-kinases (JAKs) [224]. Activated JAKs phosphorylate specific tyrosine residues on receptors, creating binding sites for cytoplasmic STAT proteins, a family of transcription factors involved in signaling from cytokine and growth factor receptors. STAT proteins, binding to phosphorylated receptors, are themselves phosphorylated by JAK, after which they dimerize and translocate into the nucleus. In the nucleus, the resulting STAT dimers bind to the promoters of target genes, regulating their transcription. In the context of COPD and tuberculosis, dysregulation of the JAK/STAT pathway may lead to an altered balance between pro- and anti-inflammatory cytokines, affecting the course of the diseases [225]. For example, activation of STAT protein variants such as STAT1 and STAT3 by IL-6 can enhance inflammation and immune response, but also contribute to inflammation chronicity and tissue damage [226].

In tuberculosis, STAT1 activation is important for antimycobacterial immunity, but over- or under-activation may impair an effective immune response [227]. In addition, some mycobacterial factors (lipoarabinomannan (LAM), 19-kDa *M. tuberculosis* lipoprotein (LpqH), ESAT-6 (Early Secreted Antigenic Target 6 kDa), CFP-10 (Culture Filtrate Protein 10 kDa), phthiocerol dimycocerosates (DIM/PDIM), secreted mycobacterial kinases and phosphatases (e.g., PtpA and PtpB)) can affect the JAK/STAT pathway, suppressing STAT activation and reducing the efficacy of the immune response [228,229]. This contributes to *M. tuberculosis* persistence and progression of infection. At the same time, chronic inflammation in COPD can lead to sustained activation of the JAK/STAT pathway, enhancing the production of pro-inflammatory cytokines and promoting tissue damage [230].

The interaction between NF-κB, MAPK, and JAK/STAT pathways is complex and multilevel. These signaling pathways can overlap and mutually regulate each other, enhancing or repressing the expression of certain genes. For example, activation of NF-κB can stimulate the expression of cytokines, which in turn activate the JAK/STAT pathway. MAPK cascades can influence the activity of NF-κB and STAT proteins through phosphorylation and alteration of their transcriptional activity [231]. Understanding these complex interactions is important for the development of novel therapeutic strategies. JAK/STAT inhibitors represent a promising therapeutic agent for the treatment of COPD by targeting key inflammatory processes and cellular mechanisms involved in disease progression. JAK inhibitors such as ruxolitinib have shown promising results in reducing cytokine production in COPD models. In particular, ruxolitinib effectively suppressed cytokine release without significantly affecting the uptake capacity of macrophages, indicating a balanced anti-inflammatory effect [232]. On the other hand, ruxolitinib has been associated with cases of tuberculosis reactivation. Several case reports indicate that patients taking ruxolitinib developed tuberculosis, including disseminated tuberculosis and tuberculous lymphadenitis [233,234,235]. Although the risk of tuberculosis reactivation with other JAK inhibitors such as tofacitinib, baricitinib, upadacitinib, and filgotinib appears to be low, long-term data are needed to fully understand the risks [236]. Although JAK/STAT inhibitors provide therapeutic benefit in a variety of diseases, their use in patients with latent tuberculosis requires careful consideration, comprehensive screening, and vigilant monitoring to reduce the risk of tuberculosis reactivation. In addition, genetic polymorphisms in the components of these signaling pathways may influence disease susceptibility and severity. The study of such genetic variations may contribute to a personalized approach to therapy and prediction of disease course.

## 7. Epigenetic Regulation of Inflammation in COPD and Tuberculosis: Role of DNA Methylation, MicroRNA and Histone Modification

In recent years, there has been growing interest in the epigenetic regulation of inflammation in various diseases, including COPD and tuberculosis [237]. Epigenetic mechanisms include DNA methylation, gene regulation through microRNA (miRNA), and histone modifications such as acetylation and deacetylation, which affect the expression of genes related to inflammation and immune response [238,239]. DNA methylation is the attachment of a methyl group to cytosine residues in DNA, usually in the CpG islands of the promoter regions of genes. This leads to conformational changes in chromatin and decreased transcriptional activity of genes [240]. In the comorbidity of COPD and tuberculosis, DNA methylation can alter the expression of key genes responsible for the regulation of inflammation and immune response [241]. Hypermethylation of promoter regions of inflammatory suppressor genes, such as IL-10 or SOCS1, leads to suppression of their expression, which contributes to enhanced inflammatory responses [242]. In addition, the methylation of genes involved in T-cell differentiation and macrophage activation may disrupt the balance between different subpopulations of immune cells, causing chronicity of inflammation and reducing the effectiveness of anti-tuberculosis immunity [243].

MiRNAs are short noncoding RNAs that regulate gene expression at the posttranscriptional level through complementary binding to target mRNAs, resulting in their degradation or inhibition of translation [244]. A large number of miRNAs have now been identified that act on a variety of targets, including regulators of the immune response and inflammation. miR-146a suppresses the expression of components of the NF-κB and MAPK signaling pathways, such as IRAK1 and TRAF6 [245]. When miR-146a expression is decreased, there is an increase in pro-inflammatory signaling, which contributes to the chronicity of inflammation in COPD and tuberculosis. In contrast, miR-155 usually acts as a pro-inflammatory factor, causing activation of macrophages and T cells, and increasing the production of cytokines such as TNF-α and IL-6 [246]. Increased expression of miR-155 may contribute to an enhanced inflammatory response, and in tuberculosis it also plays a role in the formation of effective antimycobacterial immunity [247]. An imbalance in the expression of these miRNAs in comorbidity may lead to an inadequate immune response and disease progression. The microRNA miR-145-5p is significantly reduced in lung tissues of smokers with and without COPD. It plays a protective role against cigarette smoke extract (CSE)-induced apoptosis and inflammation by targeting the KLF5 gene and modulating the NF-κB signaling pathway [248]. CSE-suppressed miR-218 regulates mucus hypersecretion and inflammation by acting on TNFR1 and suppressing NF-κB activation. Increased expression of miR-218 may ameliorate these effects, indicating its potential as a therapeutic target. Serum miR-218 levels have been found to be positively correlated with FEV1/FVC% and negatively correlated with serum IL-6 and IL-8 levels [249]. The level of miR-24-3p is decreased in COPD and is associated with increased susceptibility to apoptosis and emphysema. It regulates cellular responses to stress by affecting BIM and BRCA1, which are involved in apoptosis and DNA repair [250]. While miR-221 is suppressed in COPD patients, miR-16 and miR-146 are activated [251]. Decreased levels of miR-24-3p are associated with increased apoptosis and severity of emphysema. This microRNA regulates the expression of BIM and BRCA1, which are involved in apoptosis and DNA repair [250]. Increased expression of miR-15b is associated with areas of emphysema and fibrosis in COPD. It affects SMAD7, which is involved in the TGF-β signaling pathway [252]. Altered expression of miR-223 is observed in COPD, where it regulates monocyte-macrophage differentiation, neutrophil recruitment and pro-inflammatory responses [252,253]. MicroRNA-145-5p plays a role in regulating the immune response during tuberculosis infection. Increased expression of microRNA-145 in *M. tuberculosis*-infected macrophages suppresses cell viability and inflammation by targeting the CXCL16 gene, indicating its potential as a therapeutic target [254]. miR-145-5p can influence the immune response by modulating the expression of cytokines and other immune-related genes. For example, it has been shown to interact with the NFATc1-STAT1 regulatory pathway, which is involved in cytokine receptor binding and TNF signaling pathways [255]. miR-222 is involved in the regulation of macrophage function and immune responses, which are critical in tuberculosis. miR-222-3p has been shown to regulate the functional reprogramming of macrophages and is involved in the regulation of host innate immunity. miR-222-3p can be considered as a biological marker of tuberculosis activity [256]. It has been shown that miR-223 is upregulated in patients with active tuberculosis and that it suppresses macrophage apoptosis in tuberculosis via FOXO3, which is crucial for the immune response to *M. tuberculosis* [257].

In addition, another mechanism of epigenetic regulation is histone modifications, including histone acetylation and deacetylation, which affect chromatin structure and DNA accessibility for transcription factors [258]. Histone acetylation by histone acetyltransferases (HAT) leads to chromatin loosening and activation of gene transcription. And histone deacetylation catalyzed by histone deacetylases (HDAC) promotes chromatin condensation and suppression of gene expression [259]. In COPD, there is a decrease in HDAC activity, especially HDAC2, leading to histone hyperacetylation and increased expression of proinflammatory genes under the control of NF-κB. This contributes to increased inflammation and resistance to glucocorticosteroids [260]. In tuberculosis, MBT can affect HAT and HDAC activity in macrophages, altering the expression of genes associated with the immune response and promoting its intracellular persistence [261]. The combination of these effects in comorbidity increases pathologic inflammation and impairs the effectiveness of the immune response. Some histone deacetylase inhibitors, such as aminoacetanilide (ACE), N-Boc-1,2-phenylenediamine (N-BOC), 1,3-Diphenylurea (DFU), have shown to be effective in reducing the amount of *M. tuberculosis* in infected macrophages. These inhibitors enhance the production of antimicrobial peptides and reactive oxygen species, including β-defensin-2, LL-37, superoxide dismutase (SOD) 3, and inducible nitric oxide synthase (iNOS), which contribute to *M. tuberculosis* killing [262]. Tubastatin A, an HDAC6 inhibitor, has been shown to enhance the immune response to *M. tuberculosis* by decreasing IL-10 levels and increasing TNF-α levels, and increasing the influx of immune cells to the site of infection [263].

Thus, epigenetic changes can also affect the efficacy of pharmacotherapy [264]. Changes in DNA methylation and histone modifications can alter the expression of genes encoding drug metabolizing enzymes, transport proteins, and molecular drug targets. For example, decreased HDAC2 activity in COPD is associated with resistance to glucocorticosteroids, making it more difficult to control inflammation [265]. In tuberculosis, epigenetic regulation can affect the expression of genes involved in the response to anti-tuberculosis drugs, contributing to the development of drug resistance [266]. In turn, external factors such as smoking and exposure to air pollution can induce epigenetic changes through the generation of oxidative stress and activation of signaling pathways affecting the epigenome [267]. Oxidative stress can damage DNA and proteins, including epigenetic regulatory enzymes, leading to global changes in DNA methylation and histone modifications. This increases dysregulation of genes involved in the control of inflammation and immune response, exacerbating the course of COPD and increasing susceptibility to tuberculosis infection.

## 8. Therapeutic Perspectives on the Study of Immune Mechanisms

Better understanding of the mechanisms involved in the pathogenesis of COPD and tuberculosis has led to the identification of a wide range of potential targets for drug therapy. In addition to the well-known approaches to pharmacotherapy and the development of biologic therapies described in the previous sections, promising avenues include host-directed therapies (HDT), which include drugs aimed at modulating the body’s immune response rather than directly targeting Mycobacterium tuberculosis. Strategies include enhancing macrophage autophagy, improving cell recruitment, and balancing the cytokine response to control infection and reduce tissue damage [268,269,270,271]. Autophagy modulation aims to enhance autophagy in macrophages, which may improve the excretion of intracellular *M. tuberculosis*. This involves targeting pathways that regulate autophagy, which is critical for the destruction of intracellular pathogens [269,272]. Effects on the itaconate pathway, which involves the conversion of cis-aconitate to itaconate in macrophages, have been shown to be effective in modulating immune responses in tuberculosis and other lung diseases [273]. Inhibition of Src-tyrosine kinases may be effective in reducing *M. tuberculosis* survival in macrophages. This strategy has shown efficacy in animal models, indicating its potential as a host-directed therapy [274]. Exposure to host molecules such as cystatin F can enhance the proteolytic activity of cathepsins, leading to enhanced intracellular killing of *M. tuberculosis* in macrophages. This approach has been shown to be effective against tuberculosis, including multidrug-resistant (MDR) and extensively drug-resistant (XDR) strains [275].

One such strategy is the activation of macrophages by cytokines (IFN-γ, GM-CSF), growth factors, and synthetic substances, which enhances phagocytosis and the production of reactive oxygen and nitrogen species [276]. In this context, a promising direction, for example, is the study of the effect of vitamin B1, which contributes to the restriction of MBT growth by regulating innate immunity through PPAR-γ-dependent mechanisms [277]. In parallel, systems of targeted delivery of anti-tuberculosis drugs (nanoparticles, liposomes) are being developed using ligands that promote selective accumulation of drugs in infected macrophages [278,279]. Ursolic acid has been shown to suppress *M. tuberculosis*-induced pyroptosis and necroptosis of macrophages and to promote autophagy and enhance intracellular killing of *M. tuberculosis*, making it a potential adjuvant for tuberculosis therapy [280]. Statins, known for their cholesterol-lowering properties, have been shown to be effective in enhancing the immune response to *M. tuberculosis* by reducing cholesterol levels in macrophages, which *M. tuberculosis* uses to survive [281].

Research is also being conducted in the field of adaptive T-cell therapy, which involves genetic modification of T-lymphocytes to recognize MBT antigens and enhance their cytotoxic activity [282,283]. The modulation of T-cell subpopulations is being studied, which is aimed at restoring Th1 response and controlling regulatory cells (Th2, Th17, Treg) [284,285,286]. Attempts to use “checkpoint”-inhibitors (antibodies against PD-1/PD-L1, CTLA-4) to modulate anti-tuberculosis immunity have been described [287,288].

Dendritic cells are also being considered as targets for immunomodulation, which includes the development of vaccine platforms based on them. In these studies, their antigen-presenting activity is enhanced to generate a more effective T-cell response [289].

A promising direction is the use of cytokines (IFN-γ, IL-2, IL-7, IL-12, GM-CSF) to modulate innate and adaptive immunity, thereby enhancing the cellular response and accelerating MBT elimination [204]. Additionally, therapeutic strategies targeting regulatory myeloid cells, which can modulate the immune response in tuberculosis and potentially become a target for novel tuberculosis therapies, are being considered [290].

Genetic techniques, including CRISPR/Cas9, are being developed to modify macrophages and T lymphocytes, which may increase the resistance of these cells to intracellular proliferation of MBT [291]. Suppression of receptors used by the bacterium to invade and evade the immune response is being considered [292].

New vaccine platforms (using recombinant proteins, viral vectors, dendritic cells) are also being developed to provide more specific stimulation of anti-tuberculosis immunity, which is promising for both prevention and treatment of chronic forms of tuberculosis. Improving the efficacy of vaccines through modulation of the immune response is discussed in [293].

Thus, immunotherapy is a promising avenue for therapy of tuberculosis and COPD, but their complex overlapping immune mechanisms require better study of these mechanisms and their resulting influences.

## 9. Conclusions

COPD and respiratory tuberculosis are among the most significant medical and social problems in modern pulmonology. The widespread prevalence of these diseases, their high level of disability and mortality, as well as their serious impact on the quality of life of patients have attracted the prioritized attention of clinicians and researchers. At the same time, the importance of studying these diseases is not limited to their significance as separate nosologic units, but affects a wide range of pathophysiologic interactions that form a separate comorbid condition. The necessity to consider COPD and tuberculosis not just as separate serious diseases, but as interrelated processes, within which a specific phenotype of patients characterized by complex structural and functional changes in the respiratory system is formed, is becoming more and more obvious.

The comorbid course of COPD and tuberculosis is associated with the synergy of various complex pathogenetic mechanisms, among which activation of various inflammatory pathways and disruption of normal regulatory functions of the immune system are of key importance. Under these conditions, oxidative stress and remodeling of lung tissue increase, epithelial barrier permeability changes, cytokine balance is disturbed, and dysfunction of immune cells (neutrophils, macrophages, lymphocytes) aggravates structural changes in tissues, contributing to the progressive course of these diseases.

Despite a fairly long period of study and a significant number of studies that have increased our understanding of the pathogenesis of both diseases, many questions remain unresolved. Of interest is the evidence that *M. tuberculosis* can lead to a significant reduction in the diversity of the respiratory microbiome. Moreover, the composition of the respiratory tract microbiota allows distinguishing active tuberculosis from latent tuberculosis infection and uninfected conditions [294]. The respiratory tract microbiome is a current research topic. This is due to the fact that, for a long time, the respiratory tract was either considered sterile, or the microbiota was perceived as transient due to its ingestion with inhaled air. Thus, the understanding of the respiratory microbiota is based on the fact that it is closely related to the human immune system. Moreover, the normal composition of the respiratory tract microbiota is necessary to maintain the optimal tone of the bronchial immune system. Disruption of the normal composition of the respiratory microbiota is common in COPD, especially during exacerbations, which corresponds to an increase in local and systemic inflammation [295]. Disruption of the gut microbiota is another known problem in COPD. The gut microbiota, in addition to maintaining immune tone, is involved in the production of various substances that play important metabolic and immune regulatory roles in other organs, including the lungs. Short-chain fatty acids produced by the intestinal microbiota during fermentation of non-digestible fiber are involved in many immune mechanisms [296]. Reduction in their production plays an important role in the progression of COPD and its extrapulmonary manifestations. Nutritional support, including non-digestible fiber, is considered essential for the treatment of patients with COPD. On the other hand, pulmonary tuberculosis is also characterized by changes in the composition of the gut microbiota. The intestinal microbiota shows bilateral links with tuberculosis, which is a promising research topic [297,298,299]. The number of bacteria of the genus Prevotella from the type Bacteroidetes and bacteria of the genus Lachnospira from the type Firmicutes decreased significantly in both groups of tuberculosis patients. In addition, changes in the gut microbiota were associated with the number of CD4+ T cells in the peripheral blood of tuberculosis patients [298].

Prospective studies focusing on cellular and molecular processes, such as alveolar macrophage dysfunction or sustained immune activation, may help to clarify how chronic tuberculosis affects airway remodeling in contrast to normal COPD cases. In addition, the identification and characterization of novel biomarkers, such as specific cytokine or chemokine profiles, may provide prognostic value for disease progression and treatment efficacy in patients with COPD. This approach may lead to the development of more personalized treatment strategies for this comorbid disease. Based on these considerations, future research should focus on the detailed study of epigenetic mechanisms that influence the immune response and perpetuate chronic inflammation, the identification of molecular and cellular markers that predict COPD onset and severity, and the development of targeted immunomodulatory interventions that mitigate excessive inflammatory pathways without compromising anti-tuberculosis protection. An integrated approach to address these issues will deepen our understanding of COPD and tuberculosis comorbidity and serve as a basis for developing innovative and more targeted preventive and therapeutic strategies to improve patient outcomes and quality of life.

Thus, the study of immune mechanisms in COPD and tuberculosis, including their comorbid relationships, represents a promising field of research from the clinical point of view, the results of which may have therapeutic applications.

## Figures and Tables

**Table 1:** Studies of monoclonal antibodies in COPD.

Cytokine	Medication	Study	Result	References
IL-1β	Canakinumab	147 participants	No efficacy.	[73]
TNF-α	Infliximab	16 patients with cachexia and moderate to severe COPD	Infliximab did not induce a marked reduction in local inflammation in cachectic patients with COPD and had little effect on systemic inflammation.	[74]
		22 smokers with mild to moderate COPD	No clinically significant beneficial effects of infliximab and no significant safety concerns were observed.	[75]
		234 patients with moderate to severe COPD	Twenty-six patients (total 11.1%; placebo, 9.1%; infliximab, 12.1%) died, including nine during COPD treatment. Lung cancer was the most common type of malignancy (placebo, two cases; infliximab, ten cases).	[76]
	Etanercept	81 patients with acute exacerbation of COPD	Etanercept is no more effective than prednisone in the treatment of acute exacerbations of COPD.	[77]
IL-8	ABX-IL8	109 patients with stable COPD.	There were no significant differences in lung function, health status, distance traveled in 6 min, or side effects between groups.	[78]

**Table 2:** Role of macrophages, neutrophils, and dendritic cells in the pathogenesis of COPD and tuberculosis.

Aspect	Tuberculosis	COPD	References
Macrophages			
Primary role	Defense against *M. tuberculosis*, granuloma formation, cytokine production	Chronic inflammation, tissue damage, cytokine production	[121,122,123,124,125]
Interaction with pathogens	*M. tuberculosis* evades immune response, inhibits macrophage functions	Contribute to chronic inflammation and tissue damage	[121,122,125,126]
Macrophage polarization	M1 (initial response) and M2 (chronic infection)	M1 and M2 phenotypes depending on stage of disease	[121,122,125]
Neutrophils			
Main function	Initial immune response, phagocytosis *M. tuberculosis*	Causes pathologic changes (emphysema, mucus hypersecretion)	[127,128,129,130]
Tissue damage	Through pro-inflammatory mediators, networks and enzyme release	Through oxidative stress, networks and enzyme release	[120,128,131,132]
Dendritic cells			
Main function	Enhance host defense and manage pathogen evasion	Modulates chronic inflammation and immune response	[133,134]
Key changes	Decreased DC numbers, altered cytokine profiles	Increased pro-inflammatory markers, altered survival and migration	[135,136]

## Data Availability

The data are available from the corresponding author.

## Abbreviations

The following abbreviations are used in this manuscript:

COPD	chronic obstructive pulmonary disease
DCs	dendritic cells
IFN-γ	interferon gamma
IL	interleukin
NLRs	node-like receptors
PD-L1	programmed cell death ligand 1
TGF-β	transforming growth factor beta
Th	T-helper
TLRs	toll-like receptors
TNF-α	tumor necrosis factor-alpha
TOPD	Tuberculosis-Associated Obstructive Pulmonary Disease
Treg	regulatory T cells
